# Effects of a herbal formulation, KGC3P, and its individual component, nepetin, on coal fly dust-induced airway inflammation

**DOI:** 10.1038/s41598-020-68965-5

**Published:** 2020-08-20

**Authors:** Evelyn Saba, Young-sil Lee, Won-Kyung Yang, Yuan Yee Lee, MinKi Kim, Su-Min Woo, KilSoo Kim, Young-Sam Kwon, Tae-Hwan Kim, Dongmi Kwak, Yang-Chun Park, Han Jae Shin, Chang Kyun Han, Jae-Wook Oh, Young Cheol Lee, Hyung-Sik Kang, Man Hee Rhee, Seung-Hyung Kim

**Affiliations:** 1 Laboratory of Physiology and Cell Signalling, Department of Veterinary Medicine, College of Veterinary Medicine, Kyungpook National University, Daegu, 41566 Republic of Korea; 2 Herbal Medicine Research Division, Korea Institute of Oriental Medicine, 1672 Yuseong-daero, Yuseong-gu, Dajeon, 34054 Republic of Korea; 3 Division of Respiratory Systems, Department of Internal Medicine, College of Korean Medicine, Daejeon University, Daejeon, Republic of Korea; 4 KT&G Research Institute, Daejeon, 34128 Republic of Korea; 5 KGC Research Institute, Daejeon, 34128 Republic of Korea; 6 Institute of Traditional Medicine and Bioscience, Daejeon University, Daejeon, 34520 Republic of Korea; 7 Department of Stem Cell and Regenerative Biotechnology, Konkuk University, Seoul, 05029 Republic of Korea; 8 Department of Herbology, College of Korean Medicine, Sangji University, 83 Sangjidae-gil, Wonju, Gangwon-do 26339 Republic of Korea; 9 School of Biological Sciences and Technology, Chonnam National University, Gwangju, 500-757 Republic of Korea

**Keywords:** Cell biology, Immunology

## Abstract

Coal fly dust (CFD)-induced asthma model is used as an ambient particulate matter model of serious pulmonary damage. We aimed to evaluate the effects of a combination of ginseng and *Salvia plebeia* R. Br extract (KGC-03-PS; KG3P) and its individual components (hispidulin, nepetin and rosmarinic acid) in a CFD-induced mouse model of airway inflammation (asthma). We also evaluated signal transduction by KG3P and its individual components in the alveolar macrophage cell line, MH-S cells. In vitro, KG3P and its individual components inhibited nitric oxide production and expression of pro-inflammatory mediators and cytokines (iNOS, COX-2, IL-1β, IL-6 and TNF-α) through the NF-κB and MAPK pathways in coal fly ash (CFA)-induced inflammation in MH-S cells. Moreover, in the CFD-induced asthma model in mice, KG3P and its predominant individual component, nepetin, inhibited Asymmetric Dimethyl arginine (ADMA) and Symmetric Dimethyl arginine (SDMA) in serum, and decreased the histopathologic score in the lungs. A significant reduction in the neutrophils and immune cells in BALF and lung tissue was demonstrated, with significant reduction in the expression of the pro-inflammatory cytokines. Finally, IRAK-1 localization was also potently inhibited by KG3P and nepetin. Thus, KG3P extract can be considered as a potent candidate for amelioration of airway inflammation.

Presently, air pollution is a serious environmental problem encountered by living organisms. Extensive industrialization and a sedentary life style, aggravated by the increased usage of automobiles than the physical methods of transportation, are the etiological factors associated with this problem^1^. Increased usage of automobiles and the installation of industrial units near urban areas are directly related to increased production of smoke, which consists of ambient particulate matters such as coal, asbestos, and combustion particles. Although acute inhalation of these nanoparticles does not pose any serious side effects to health, constant and chronic exposure can lead to the settling of these particles in the respiratory tract, resulting in serious chronic pulmonary aliments such as asthma, chronic bronchitis, and lung cancer^2–4^. Industrial combustion is considered a well-studied process and the fly ash produced by combustion is frequently used as a model particle for studying fine and nano-fine components in toxicological research^5,6^. Since these fine particles can cause chronic, fatal pulmonary diseases, there has been a shift in research focus towards the discovery of remedies (both allopathic and herbal).

Ginseng, popularly considered a panacea, has been used for centuries in the Korean peninsula owing to its outstanding health promoting effects against minor to major diseases, including inflammation and cancer^7–9^. Extensive data are available on the health enhancing properties of whole ginseng extract as well as for the individual compounds in its extract, termed ginsenosides^10^. To date, however, no data exists on the effect of ginseng in coal fly ash (CFA) or combustion particle-induced inflammation.

*Salvia plebeia* R BR (SPR-Br) is a common herbal medicine used in China and is often used to treat urinary tract infections. SPR-Br is known to possess anti-inflammatory, anti-oxidant, anticancer, anti-hypertensive, and immune boosting effects^11,12^. The plant is found near streams and mountains that are $>$ 2,800 m above sea level^13^. SPR-Br is composed of flavones, organic acids, amino acids, and alcohol phenols^14^. Most importantly, it consists of many single compounds such as hispidulin, nepetin, eupafolin, ursoic acid, and caffeic acid^15^. Owing to previous reports, SPR-Br has been confirmed to possess strong biological properties; however, there is no data available on the effects of this herb on airway inflammation induced by combustion particles.

We aimed to develop a herbal formulation consisting of ginseng extract and SPR-Br (KG3P) to verify the effects of the extract and its individual components on MH-S alveolar macrophage cells induced by CFA, and mice with airway inflammation induced by coal fly dust (CFD). Based on the HPLC analysis performed, KG3P and its three most abundant components potently suppressed inflammation by decreasing the levels of nitric oxide (NO), iNOS, COX-2, IL-1β, IL-6, and TNF-α via the NF-κB and MAPK pathways in MH-S cells. Moreover, our in vivo results showed that KG3P and its component, nepetin, downregulated the inflammatory cytokines in BALF and lung tissues, and decreased the fluorescent intensity of interleukin-1 receptor associated kinase 1 (IRAK-1) in lung tissues. These results suggest that this mixture can be used as an excellent remedy in the prevention of pulmonary disorders caused by air pollution.

## Results

### Effects of KG3P and nepetin in vitro on MH-S cells and signal transduction via the NF-κB and MAPK pathways

**Figure 1:**
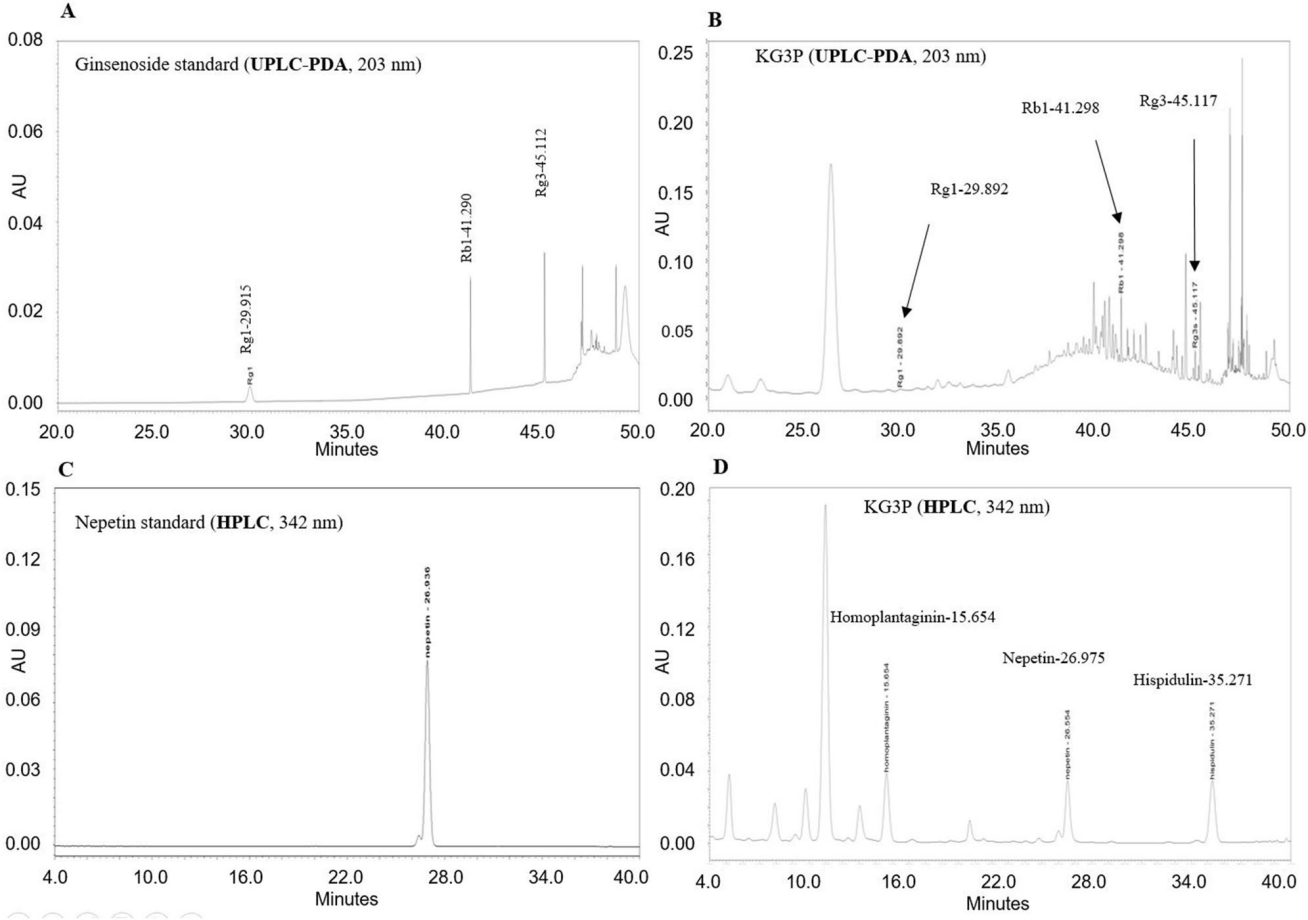
Chromatogram of four compounds purified from the KGC-03-PS mixture (KG3P). Rg1, Rb1, Rg3s, and nepetin were identified as marker compounds of the Korean Red Ginseng and *Salvia plebeia* R. Br. mixture (KG3P). (**A**) UPLC-PDA chromatograms of three standard ginsenoside mixtures at 203 nm. (**B**) UPLC-PDA chromatogram for KG3P mixture at 203 nm. (**C**) HPLC chromatograms for standard nepetin at 342 nm and (**D**) HPLC chromatograms for KG3P mixture at 342 nm. Rg1 (0.22 mg/g ± 0.03), Rb1 (2.06 mg/g ± 0.06), Rg3 (0.51 mg/g ± 0.05), and nepetin (5.42 mg/g ± 0.39) appeared at a retention time of approximately 29.8, 41.2, 45.1, and 26.9 min, respectively. *UPLC-PDA* ultra-performance liquid chromatography-photodiode array detector, *HPLC* high-performance liquid chromatography.

**Figure 2:**
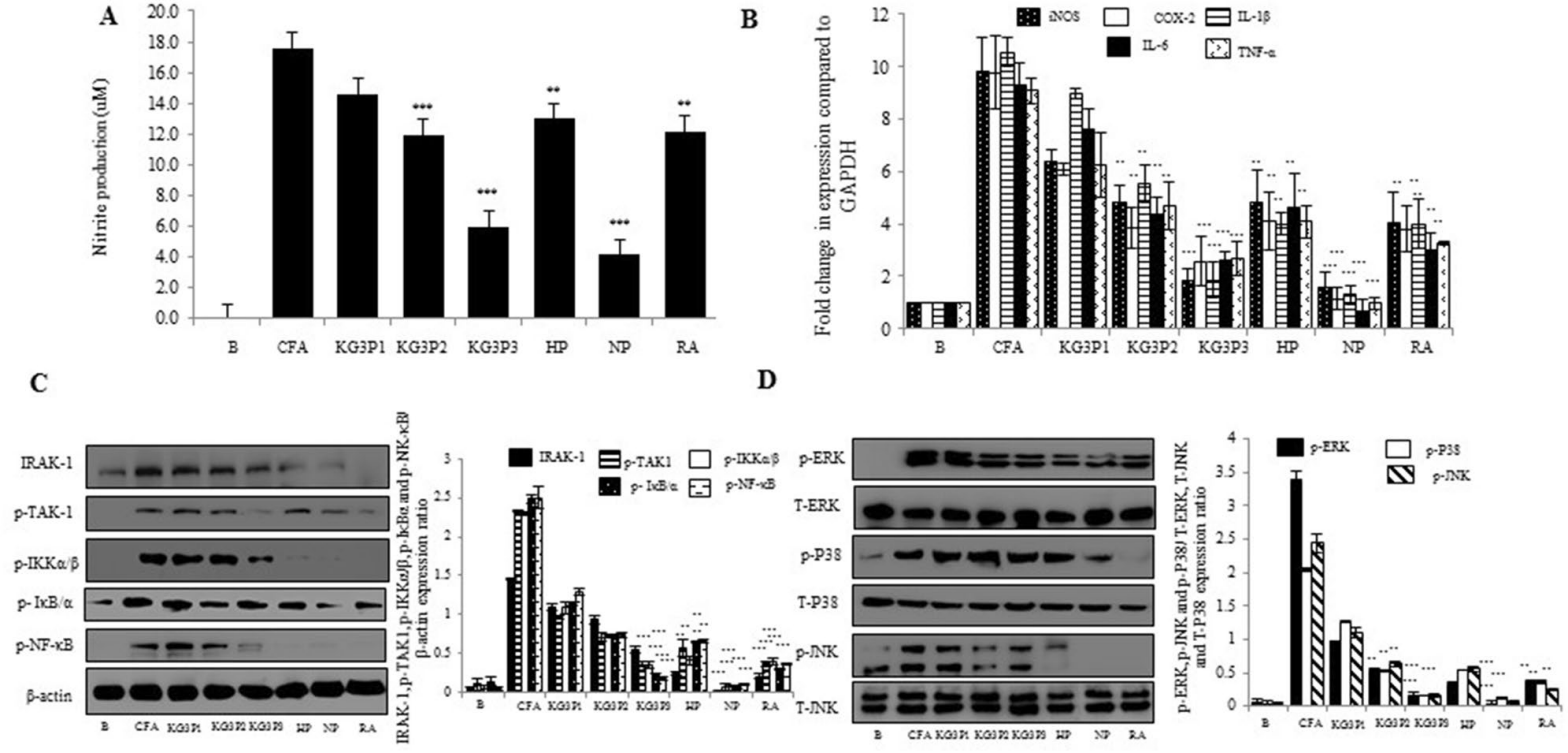
Effects of KG3P and nepetin in vitro on MH-S cells and signal transduction via the NF-κB and MAPK pathways. (**A**) Nitrite production was inhibited by dose dependent concentrations of KG3P (25, 50 and 100 μg/mL) and hispidulin (HP; 20 μM), nepetin (NP; 20 μM) and rosmarinic acid (RA; 20 μM) as determined by Griess method. Values in the bar graphs represent means ± SEM of three independent experiments. ***p < 0.001 and **p < 0.05 are considered significant compared to the CFA-only group. (**B**) Real-Time PCR for KG3P and single compounds. Values in the bar graphs represent means ± SEM of three independent experiments. ***p < 0.001 and **p < 0.05 are considered significant compared to the CFA-only group. (**C**, **D**) Signal transduction of KG3P and single compounds through the NF-κB and MAPK pathways in MH-S cells via western blot analysis. Values in the bar graphs represent means ± SEM of three independent experiments. ***p < 0.001 and **p < 0.05 are considered significant compared to the CFA-only group. *KG3P1* KG3P 25 μg/mL, *KG3P2* KG3P 50 μg/mL, *KG3P3* KG3P 100 μg/mL, *B* Basal, *CFA* Coal Fly ash, *HP* Hispidulin, *NP* Nepetin and *RA* Rosmarinic acid. Full length western blots are shown in Supplementary Fig. 2a,b.

Based on our UPLC results as shown if Fig. 1, KG3P possessed three abundant individual components: hispidulin, nepetin, and rosmarinic acid. Before we start with the in vivo work, we sought to elucidate the in vitro effects of various concentrations of KG3P in Alveolar macrophage cells (MH-S cells) stimulated with CFA. We selected alveolar macrophage cells for our study because in case of asthma induction, it’s the airway inflammation that is responsible for the pathophysiology, and since macrophage cells are the major players in this process^16,17^ therefore we checked their levels in response to asthma induction and treatment with KG3P and nepetin along with the positive control Montelukast. As shown in Fig. 2A,B, KG3P potently inhibited nitrite production and expression of pro-inflammatory mediators and cytokines in a dose dependent manner; the individual components, especially nepetin, also exhibited this potent inhibition. In addition, the highest dose of KG3P and nepetin, mainly, inhibited the phosphorylation of all downstream factors in the NF-κB and MAPK pathways in MH-S cells (Fig. 2C,D). Since nepetin was demonstrated as the most abundant and effective of the three individual components, the in vivo study was performed using only nepetin and KG3P.

### Inhibitory effects of KG3P and nepetin on serum asymmetrical dimethyl arginine (ADMA) and symmetric dimethyl arginie (SDMA) levels, and restoration of histopathological lesions

**Figure 3:**
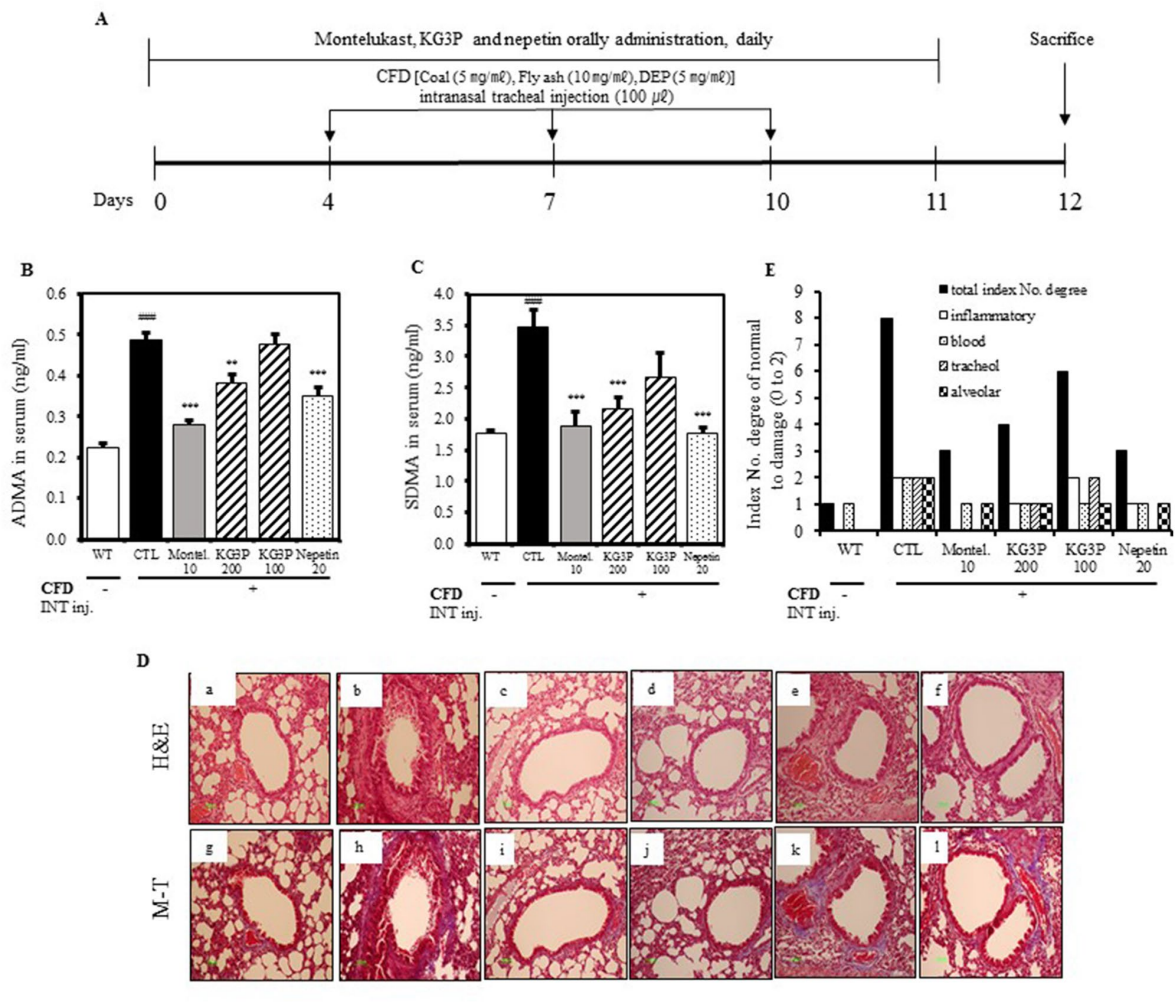
Inhibition of airway inflammation by the KG3P and nepetin in a CFD-induced murine model of airway inflammation. (**A**) Scheme for the CFD sensitization and challenge protocol. Mice were exposed to 100 μL of CFD [Coal (5 mg/mL), Fly ash (10 mg/mL), Diesel exhaust particles (DEP, 5 mg/mL)] mixed solution by intranasal tracheal injection thrice in 3 day intervals for 12 days. (**B**, **C**) KG3P and nepetin inhibited asymmetric dimethyl-arginine (ADMA) and symmetric dimethly-arginine (SDMA) production in serum obtained from CFD mice by ELISA kit. (**D**) Effect of KG3P and nepetin treatment on lung histopathology in CFD-CTL mice as visualized by H&E and Masson’s Trichrome staining. . Representative sections from each treatment group are shown. (a) BALB/c normal Wild type control (WT), (b) CFD-sensitized control mice (CTL), (c) 10 mg/kg montelukast-treated CFD-sensitized mice, (d) 200 mg/kg KG3P-treated CFD-sensitized mice, (e) 100 mg/kg KG3P-treated CFD-sensitized mice, and (f) 20 mg/kg nepetin-treated CFD-sensitized mice. M–T staining pictures have the same order for groups in H&E staining (g–l). (**E**) Quantitative analyses of the degree of lung tissue damage in the sections. Data are from individual mice, with arithmetic mean points shown in histograms. Values are expressed as mean ± SEM (n = 8 mice). ^#^*p* < 0.05, ^##^*p* < 0.01, and ^###^*p* < 0.001 (compared to WT), and *p < 0.05, **p < 0.01, and ***p < 0.001 (compared to CTL).

Asymmetrical dimethyl arginine (ADMA) and symmetric dimethyl arginie (SDMA) are involved in the inflammation, endothelial dysfunction and oxidative stress. Basically they are the structural analogues of l-arginine, which competitively regress NO synthase, ultimately leading to decreased basal NO production with the fact that basal NO production is essential for cellular proliferation, vasodilation and migration^18–20^. Therefore we had checked the effects of the KG3P treatment on the serum ADMA and SDMA levels. As shown in Fig. 3B,C, both ADMA and SDMA were potently reduced by the positive control, montelukast, and by higher doses of KG3P and nepetin. As observed in Fig. 3D,E, higher doses of KG3P and nepetin restored the histology of lungs toward normal and decreased the histopathological score.

### Decreased number of immune cells in BALF and lung tissue

**Figure 4:**
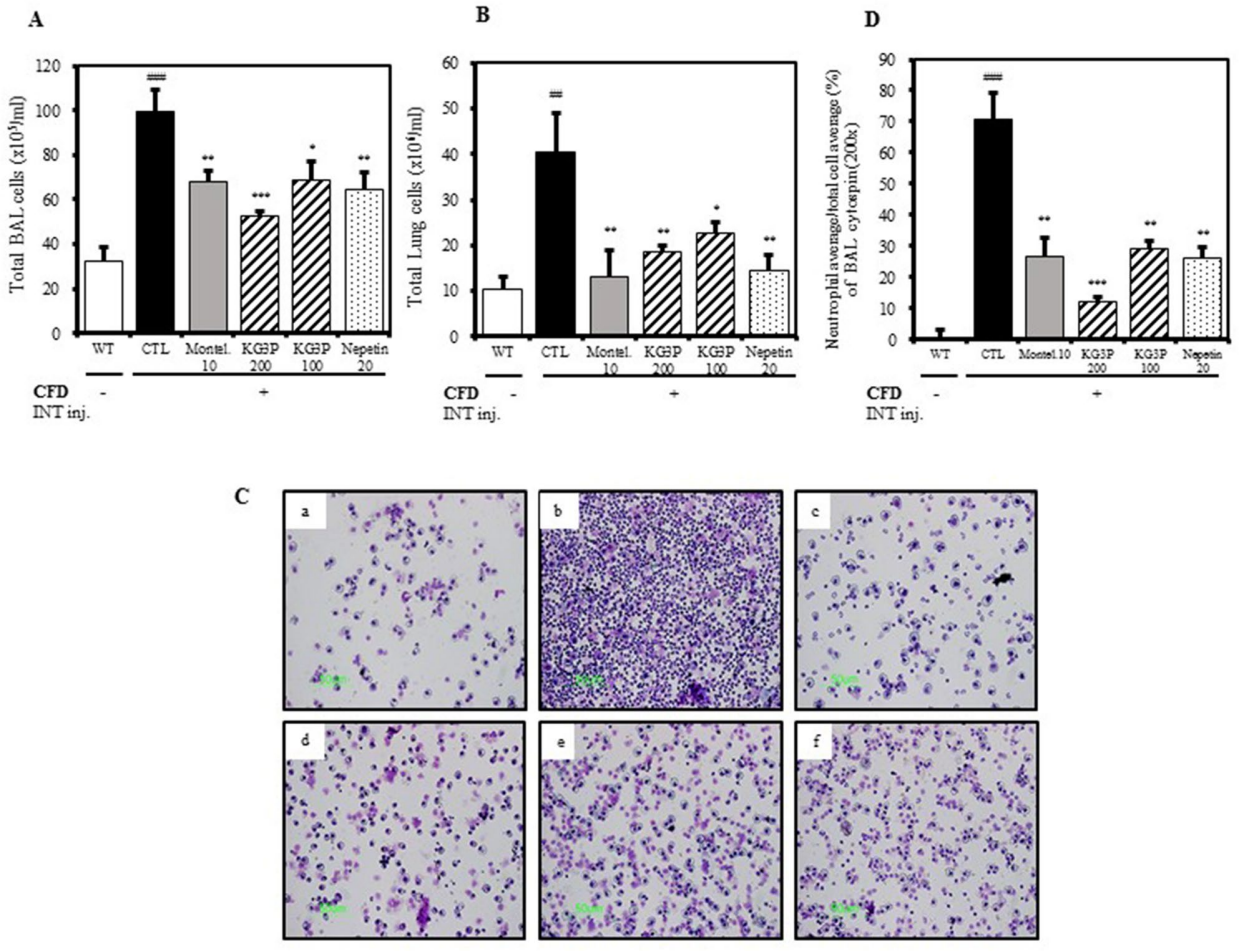
The effects of KG3P and nepetin on airway immune cell number and neutrophilic airway inflammation in CFD-induced airway inflammation murine model. (**A**) Total BALF cells from each treatment group. (**B**) Total lung cells from each treatment group. For A&B, cells were counted using a haemocytometer. (**C**) Photomicrograph of BALF cytospins from mice with CFD-induced airway inflammation (magnification, ×200), (a) BALB/c normal Wild type control (WT), (b) CFD-sensitized control mice (CTL), (c) 10 mg/kg montelukast-treated CFD-sensitized mice, (d) 200 mg/kg KG3P-treated CFD-sensitized mice, (e) 100 mg/kg KG3P-treated CFD-sensitized mice, and (f) 20 mg/kg nepetin-treated CFD-sensitized mice. (**D**) KG3P and nepetin suppressed neutrophilia in BALF. Data are from individual mice, with arithmetic mean points shown in histograms. Values in bar graphs are expressed as mean ± SEM (n = 8 mice). ^#^*p* < 0.05, ^##^*p* < 0.01, and ^###^*p* < 0.001 (compared to WT), and *p < 0.05, **p < 0.01, and ***p < 0.001 (compared to CTL).

**Figure 5:**
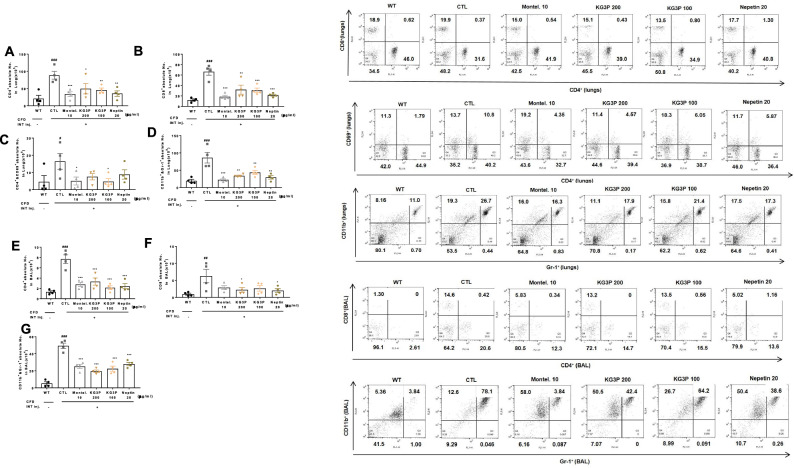
Reduction in the numbers of Immune cell subtypes in BALF and Lung cells by Fluorescent activated cell sorting analysis (FACS). (**A**, **E**) Lung and BALF cells were stained with anti-CD3^+^/CD4^+^ in lung and BALF, (**B**, **F**) anti-CD3^+^/CD8^+^ in lung and BALF, (**C**) CD4^+^/CD69^+^, (**D**, **G**) Gr-1^+^/CD11b^+^ in lung and BALF, mAbs. (BALB/c normal Wild type control (WT), CFD-sensitized control mice (CTL), 10 mg/kg montelukast-treated CFD-sensitized mice (Montel. 10), 200 mg/kg KG3P-treated CFD-sensitized mice (KG3P 200), 100 mg/kg KG3P-treated CFD-sensitized mice (KG3P 100), and (20 mg/kg nepetin-treated CFD-sensitized mice (Nepetin. 20). Values in bar graphs are expressed as mean ± SEM (n = 8 mice). ^#^*p* < 0.05, ^##^*p* < 0.01, and ^###^*p* < 0.001 (compared to WT), and *p < 0.05, **p < 0.01, and ***p < 0.001 (compared to CTL).

Generally, there is an increase in immune cells during the invasion of foreign particles in the body which is the natural adaptive immune response^21^. We therefore sought to check the immune cell levels in the lungs and BALF. As shown in Fig. 4A‒D, montelukast, both doses of KG3P, and nepetin potently suppressed the number of total immune cells and neutrophils in BALF and lung samples. Moreover, using FACS analysis (Table 1 and Fig. 5, A–G), CD4^+^, CD8^+^, and CD11b^+^ cells were significantly decreased in BALF and lungs cells, indicating that the over activation of the immune system caused by CFD was positively suppressed by the higher doses of KG3P and nepetin, decreasing the aggravation of inflammation.

**Table 1:** Fluorescence-activated cell sorting analysis (FACS) of immune cell subtypes in lung and BALF.

Cell phenotypes in Lung, BALF		CFD-induced airway inflammation murine model (Absolute No.)					
		Normal BALB/c (WT)	CTL 0	Montel. (10 mg/kg)	KG3P (200 mg/kg)	KG3P (100 mg/kg)	Nepetin (20 mg/kg)
CD3^+^/CD4^+^ (× 10^6^ cells)	Lung	9.8 ± 4.73	79.1 ± 9.5^###^	27.6 ± 12.1**	29.4 ± 5.05***	37.1 ± 0.55***	25.0 ± 8.89***
CD3^+^/CD8^+^ (× 10^5^ cells)		12.5 ± 1.40	73.3 ± 4.16^###^	15.6 ± 0.82***	25.7 ± 6.13***	25.1 ± 1.66***	21.5 ± 0.97***
CD4^+^/CD69^+^ (× 10^5^ cells)							
		0.4 ± 0.02	9.10 ± 2.65^##^	1.40 ± 0.37**	4.80 ± 2.21	2.70 ± 0.69*	5.40 ± 1.03
Gr-1^+^/CD11b^+^ (× 10^5^ cells)		16.6 ± 3.1	78.2 ± 16.4^##^	23.5 ± 6.9**	32.6 ± 0.57**	36.3 ± 6.2*	33.7 ± 6.5**
CD3^+^/CD4^+^ (× 10^4^ cells)	BALF	1.30 ± 0.22	9.0 ± 0.36^###^	3.4 ± 0.11***	3.5 ± 1.54***	2.4 ± 0.46***	2.8 ± 1.01***
CD3^+^/CD8^+^ (× 10^4^ cells)		1.40 ± 0.17	6.7 ± 2.43^#^	2.8 ± 0.09	2.8 ± 1.40	2.5 ± 1.49	2.1 ± 1.30
Gr-1^+^/CD11b^+^ (× 10^4^ cells)		2.10 ± 0.25	53.6 ± 3.12^###^	22.5 ± 4.49***	21.5 ± 1.94***	20.9 ± 2.78***	26.4 ± 3.04***

Each point represents the mean ± SEM values for 6 mice.

^#^*p* < 0.05, ^##^*p* < 0.01, and ^###^*p* < 0.001 (compared to WT), and *p < 0.05, **p < 0.01, and ***p < 0.001 (compared to CFD).

### Suppression of pro-inflammatory cytokines expression in BALF and lung tissue

**Figure 6:**
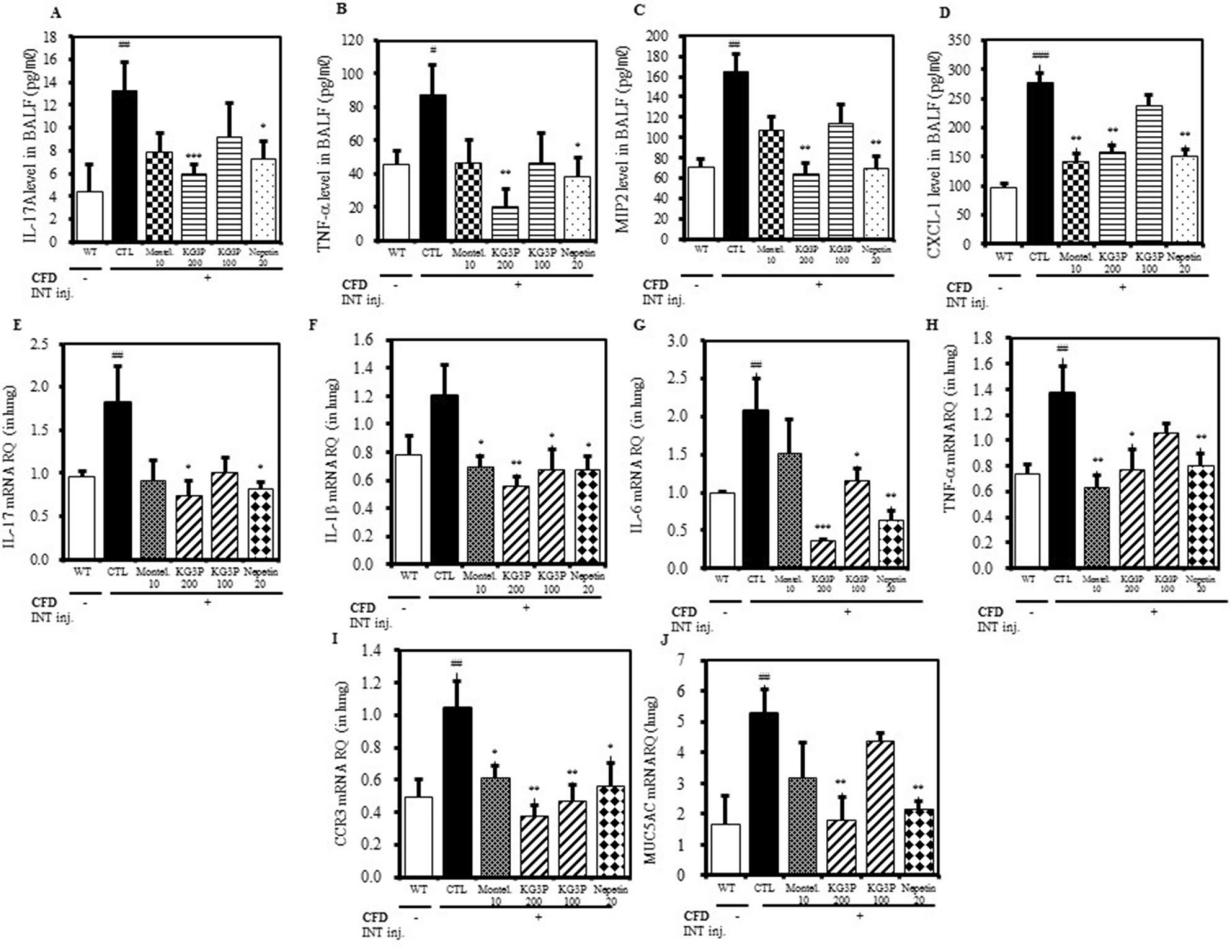
The effects of KG3P and nepetin on pro-inflammatory cytokines in BALF and lung tissue. KG3P and nepetin reduced (**A**) IL-17A, (**B**) TNF-α, (**C**) MIP2, (**D**) and CXCL1 levels in the BALF obtained from serum collected from WT, CFD, Montelukast (10 mg/kg), KG3P (200 mg/kg, 100 mg/kg), and nepetin (20 mg/kg) treated mice; these levels were measured using ELISA. (**E**) Effects of KG3P and nepetin on the expression of cytokine mRNA in lung tissues. IL-17, (**F**) IL-1β, (**G**) IL-6 (**H**) TNF-α, (**I**) CCR3, (**J**) and MUC5AC expression was determined by Real-Time PCR. Expression is presented as relative quantitation (RQ). Data are from individual mice, with arithmetic mean points shown in histograms. Values in bar graphs are expressed as mean ± SEM (n = 8 mice). ^#^*p* < 0.05, ^##^*p* < 0.01, and ^###^*p* < 0.001 (compared to WT), and *p < 0.05, **p < 0.01, and ***p < 0.001 (compared to CTL).

Pro-inflammatory cytokines are secreted in response to inflammation and timely control is essential; this is because an uncontrolled secretion of these chemicals can irreversibly damage the tissue^22^. And it is a renowned fact that the major contributors in the pathology of asthma are the cytokines that are released from lung cells. Keeping this in mind, we had investigated the levels of these pro-inflammatory cytokines in the BALF and lung tissue. From Fig. 6A‒C, the higher doses of KG3P and nepetin are shown to significantly suppress the levels of IL-17,which is naturally elevated in asthma causing allergic rhinitis^23^, TNF-α, which is found to be potently involved in many aspects related to airway pathology in asthma and which is also one of the target for asthma treatment^24^, and MIP2 which is activated by IL-17^25^. In addition to these, CXCL-1 which attracts neutrophils to the sites of airway inflammation^26^ in BALF samples from mice was also downregulated by KG3P treatment (Fig. 6D). Moreover, montelukast, both doses of KG3P, and nepetin significantly inhibited the expression of IL-17, IL-1β, IL-6 and TNF-α which are considered to be the top prioritized pro-inflammatory agents^27^ (Fig. 6E–H). Lastly, CCR3 which is present on the T-cells co-localizing with eosinophils in the allergic asthma condition^28^, and MUC5AC which is the major component of mucous causing airway obstruction in asthma^29^ (Fig. 6I,J) were also ameliorated by KG3P and nepetin, indicating that the extract and the individual component are indeed potent anti-inflammatory agents.

### Inhibition of fluorescence intensity of IRAK-1 and signal transduction of KG3P and nepetin via the NF-κB and MAPK pathways

**Figure 7:**
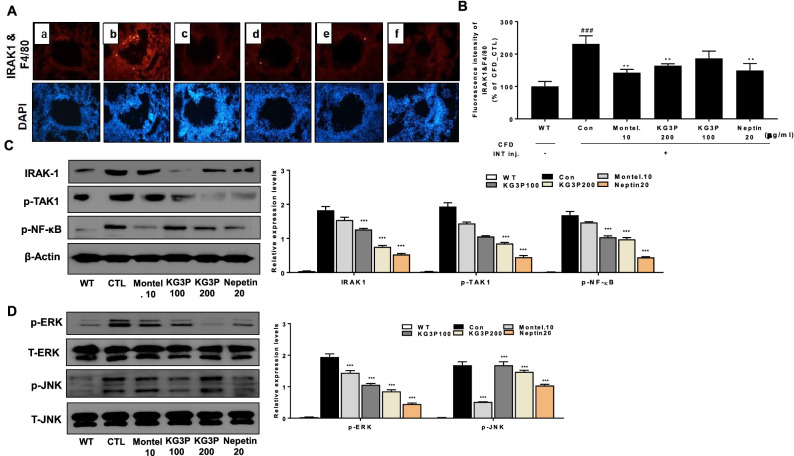
Immunohistofluorescence (IHF) staining of IRAK1 and signal transduction of KG3P and nepetin via the NF-κB and MAPK pathways in lung tissue. (**A**) IRAK1 (green), F4/80 (red) and DAPI (DNA) (grey) in lung tissue as determined from Immunofluorescence microscope. (a) BALB/c normal Wild type control (WT), (b) CFD-sensitized control mice (CTL), (c) 10 mg/kg montelukast-treated CFD-sensitized mice, (d) 200 mg/kg KG3P-treated CFD-sensitized mice, (e) 100 mg/kg KG3P-treated CFD-sensitized mice, and (f) 20 mg/kg nepetin-treated CFD-sensitized mice. (**B**) Data are from individual mice, with arithmetic mean points shown in bar graphs. Values in bar graphs are expressed as mean ± SEM (n = 8). ^#^*p* < 0.05, ^##^*p* < 0.01, and ^###^*p* < 0.001 (compared to WT), and *p < 0.05, **p < 0.01, and ***p < 0.001 (compared to CTL). (C-D) Signal transduction of KG3P and nepetin through the NF-κB and MAPK pathways in lung tissue in the CFD mice airway inflammation model by western blotting. Values in the bar graphs are presented as mean ± SEM of three independent experiments. In the bar graphs and blots, BALB/c normal Wild type control (WT), CFD-sensitized control mice (CTL), 10 mg/kg montelukast-treated CFD-sensitized mice (Montel. 10), 200 mg/kg KG3P-treated CFD-sensitized mice (KG3P 200), 100 mg/kg KG3P-treated CFD-sensitized mice (KG3P 100), and 20 mg/kg nepetin-treated CFD-sensitized mice (Nepetin 20). ***p < 0.001 and **p < 0.05 are considered significant compared to the CTL group. Full length blots are shown in Supplementary Fig. 2c,d.

Interleukin 1 receptor associated kinase (IRAK-1) is the negative regulator of Toll like receptor 1 and it is key activator of NF-κB and MAPK pathways. In fact, continuous activation of IRAK-1 causes the onset of persistent asthma in humans^30^. We therefore checked lung tissues which were stained with IRAK-1 antibody to visualize the presence of IRAK-1 positive cells with KG3P and nepetin. As shown in Fig. 7A,B, IRAK-1 positive cells were potently inhibited in the lung tissue by montelukast, both doses of KG3P, and nepetin. This was also confirmed via western blot analysis of lung tissue protein where the higher dose of KG3P and nepetin potently inhibited the expression of IRAK-1, p-TAK1, p-NF-κB, p-ERK, and p-JNK (Fig. 7C,D). These results strongly suggest that KG3P and the single compound, nepetin, exhibit their anti-inflammatory effects via the NF-κB and MAPK pathways during CFD-induced airway inflammation.

## Discussion

Numerous epidemiological studies are available on the effects of fine and ultrafine particulate matter (PM) on health, especially those relating to pulmonary disorders that in the long-term, lead to extensive morbidity and mortality. The composition of PM include coal, ash, oil, and diesel exhaust particles (DEP)^31,32^. The incidence of chronic pulmonary diseases, especially lung cancer, that occur due to chronic deposits of these fine particles in tissues over a long period of time, is a major concern of scientists^2^. The current lifestyle trend inevitably exposes living organisms to the inhalation of these particles; thus, many scientists are attempting to devise various preventive and therapeutic remedies (of chemical and herbal origin) to avoid incidence of PM-induced chronic diseases.

Few studies exist on the effects of natural herbal extracts or compounds on CFD-induced airway inflammation using a mice model. However, several studies have been performed using lung epithelial cells to examine the effects of CFD on reactive oxygen species or pro-inflammatory cytokines^33^. A study reported that iron, which constitutes up to 14% of CFA, was responsible for the expression and secretion of the pro-inflammatory cytokine, IL-8, in A549 human lung epithelial cells^34^. Another study examined the initiation and secretion of reactive oxygen species and cytokines, primarily IL-6, TNF-α, and IL-8 in human bronchial epithelial cells (BEAS-2B) and RAW 264.7 cells exposed to CFA or PMs^35^. Similar to this study, we found increased expression of NO, iNOS, COX-2, and pro-inflammatory cytokines such as IL-1β, IL-6, and TNF-α in the murine macrophage MH-S cell line when stimulated with CFA. However, as expected, these elevated levels of NO and pro-inflammatory mediators and cytokines were decreased by treatment with KG3P and its individual components (Fig. 2A,B).

Based on the mechanistics, various studies have shown that these PMs can stimulate toll like receptor (TLR) 2 and 4. Once these particulate matters stimulate TLR4 receptors, the classical NF-κB pathway is mainly activated. This includes the activation of factors downstream of TLR4, which includes IRAK-1, transforming growth factor beta-activated kinase 1 (TAK-1), etc., which ultimately causes translocation of NF-κB from the cytoplasm to the nucleus to activate the NF-κB pathway^36^. Certainly, several studies have shown that in alveolar macrophage cells, treatment with a TLR4 antagonist results in minimal release of pro-inflammatory cytokines^37^. Importantly, in our study, CFA activated the NF-κB and MAPK pathways, both of which are primarily activated during foreign invasion, in this case the PMs relative to previously reported studies^38, 39^. We hypothesize that CFA may bind to TLR4 receptors, as shown in Fig. 2C,D, as all downstream factors for NF-κB [mainly IRAK-1 and TAK-1, and MAPK (ERK, JNK and P38)] were activated by CFA and simultaneously inhibited by KG3P and the individual components. Among the individual components identified in the KG3P extract, nepetin was the most abundant. Previous reports on the biological activities such as the anti-microbial, anti-oxidant, anti-tumour, and anti-inflammatory of nepetin have been presented; however, its effect on airway inflammation or asthma remained un-investigated^40–42^. Therefore, we continued our in vivo study with nepetin from the KG3P extract.

Many studies have reported the effects of either PMs, CFA, or CFD in various murine and rodent models of pulmonary ailments such as asthma and airway inflammation^43^. Walters et al*.*^44^ reported that PMs induced an increase in the amount of immune cells in BALF and lung cells and increased the expression of pro-inflammatory cytokines in lung tissues. Takano et al*.*^45^ reported that the inhalation of DEP exacerbates allergen-related eosinophil recruitment and airway hyper-responsiveness in mice. Similar to these studies, we found that exposure of mice to CFD resulted in elevated levels of ADMA and SDMA (both of which are analogues of NO during inflammation), causing histological changes in lung tissues including infiltration of inflammatory cells, collagenous fibre production, and increased mucous production due to downstream secretion of pro-inflammatory cytokines such as interleukins and TNF-α. Various immune cells in BALF and the amounts of neutrophils in lung tissues (Figs. 3, 4, 5) were positively inhibited by KG3P and nepetin, respectively.

We also observed elevated expression of pro-inflammatory cytokines, primarily the interleukins (i.e., IL-17, IL-1β, IL-6, and TNF-α) (Fig. 6) in BALF and lung tissues; this is similar to previous studies that indicated the activation of pro-inflammatory cytokines during airway inflammation and our in vitro results using the CFA-induced MH-S cells^46^. Macrophage inflammatory protein-2 (MIP2), chemokine (C-X-C motif) ligand 1 (CXCL1), and CC- chemokine receptor 3 (CCR3) are major inflammatory factors expressed in airway inflammation and asthma^47–51^. In addition, polymeric mucin gene (MUC5AC) is expressed and its activation causes disruption in the secretion and storage of mucin, thus leading to mucus gel formation. This mucus gel obstructs the airway epithelium resulting in asthma and bronchitis^29,52^. In our mouse model, these factors were elevated; however, as expected, oral treatment with KG3P and nepetin significantly decreased the levels of these parameters, and the effects were comparable to those observed on treatment of mice with the positive control, montelukast (administered to treat asthma^53^). This indicates that the decreased levels of expression of these genes inhibited the infiltration of inflammatory cells and decreased the thickness of the airway epithelium. We also confirmed the signal transduction of KG3P and nepetin via the IRAK-1-NK-κB pathway using immunohistofluorescence and immunoblot experiments. KG3P and nepetin potently inhibited the localization of IRAK-1 in lungs and inhibited the phosphorylation of IRAK-1, p-TAK-1 and NF-κB in lungs tissue, and ERK and JNK from the MAPK pathways (Fig. 7). These results confirm the in vitro data obtained using the CFA-induced MH-S cells.

Therefore, we demonstrated the novel anti-inflammatory/anti-asthmatic effects of a herbal formulation with KG3P and those exhibited by its individual component, nepetin, on murine alveolar macrophage MH-S cells and CFD-induced airway inflammation mouse model. Using an even greater mechanistic study, this formulation may be proven to be a promising herbal remedy for the prevention of PM-/CFA-/DEP-/CFD-induced airway ailments.

## Materials and methods

### Chemicals and reagents

The detailed chemicals and reagents are given in Supplementary information 1.

### Sample preparation for KGC-03-PS (KG3P)

Korean red ginseng extract powder was procured from Korea Ginseng Corporation. Dried SPR-Br was purchased from Dongjin Farm (Buan, Jeonbuk, Korea) and extracted twice with a 15-fold volume of 30% alcohol/water for 4 h at 80 ℃. Thereafter, the extract was filtered through a 1 µm pore size, and the resultant mixture concentrated using an evaporator prior to preparing the powdered form by spray-drying. KGC-03-PS (KG3P) was prepared by a mixture of Korean red ginseng:SPR-Br, (1:3). High performance liquid chromatography (HPLC) was then used to analyse the mixture and the following composition obtained: Ginsenoside Rg1 (0.22 ± 0.03 mg/g), Rb1 (2.06 ± 0.06 mg/g), 20(*S*)-Rg3 (0.51 ± 0.05 mg/g), and nepetin (5.42 ± 0.39 mg/g) using standard operating procedure.

Ginsenoside Rg1, Rb1, and 20(*S*)-Rg3 standards were purchased from Chromadex (Irvine, USA) and 2 g of KGC-03-PS powder extracted with 10 ml of 30% aq. methanol in an ultrasonic chamber for 30 min. After ultrasonic extraction, centrifugal separation (Legand Mach 1.6R, Thermo, Germany) was performed for 10 min at 3,000 rpm. The resulting supernatant solution was filtered (0.2 µm, Acrodisk, USA) and injected into the UPLC-PDA system (Waters Co., USA).

The instrumental conditions of UPLC-PDA were as follows: the chromatographic separation was obtained using a BEH C18 column (100 mm × 2.1 mm, 1.7 µm, Waters Co., USA) and the column temperature set to 40 °C. The binary gradient elution system consisted of 0.001% phosphoric acid in water (A) and 0.001% phosphoric acid in acetonitrile (B). Separation was achieved using the following protocol: 0–25.0 min (15% B), 35.0 min (20% B), 39.5 min (30% B), 40.5 min (32% B), 42.5 min (40% B), 46.0 min (65% B), 47.0–49.0 min (90% B), and 51.0–53.0 min (15% B). The flow rate and sample injection volume were set to 0.6 mL/min and 2.0  µL, respectively. The ginsenosides were determined at a UV wavelength of 203 nm using a photodiode array detector (Waters Co., USA).

Nepetin standard was purchased from Avention (Seoul, Korea). Half gram of KGC-03-PS powder was extracted with 20 mL of methanol in an ultrasonic chamber for 30 min, and diluted to 50 mL with methanol. After extraction, the sample solution was filtered (0.2 µm, Acrodisk, USA) and injected into the HPLC–PDA system (Waters Co., USA).

The instrumental conditions of HPLC–PDA were as follows: chromatographic separation was performed using a Zorbax eclipse XDB C18 column (250 mm × 4.6 mm, 5 µm, Agilent Co., USA). The binary gradient elution system consisted of 0.05% acetic acid in water (A) and methanol (B). Separation was achieved using the following protocol: 0.0 min (38% B), 14.0 min (42% B), 17.0 min (45% B), 17.1 min (48% B), 32.0 min (50% B), 40.0 min (85% B), 45.0 min (38% B). The flow rate and sample injection volume were set to 1.0 mL/min and 10.0 µL, respectively. Nepetin was determined at a UV wavelength of 342 nm using a photodiode array detector (Waters Co., USA). All HPLC chromatograms are presented in Fig. 1A‒D.

### Cell culture

The detailed method is given in Supplementary information 1.

### Nitric oxide (NO) assay

The detailed method is given in Supplementary information 1.

### Cell Viability (MTT) assay

The detailed method is given in Supplementary information 1.

### RNA extraction and polymerase chain reaction (PCR)

The detailed method is given in Supplementary information 1 (Table 2).

**Table 2:** Primer sequences for RT-PCR and real-time PCR.

Gene	Primer	Oligonucleotide sequence (5′–3′)
GAPDH	F	5′-CAATGAATACGGCTACAGCAAC-3′
	R	5′-AGGGAGATGCTCAGTGTTGG-3′
iNOS	F	5′-CCCTTCCGAAGTTTCTGGCAGCAGC-3′
	R	5′-GGCTGTCAGAGCCTCGTGGCTTTGG-3′
COX-2	F	5′-TCTCAGCACCCACCCGCTCA-3′
	R	5′-GCCCCGTAGACCCTGCTCGA-3′
IL-1β	F	5′-CAGGGTGGGTGTGCCGTCTTTC-3′
	R	5′-TGCTTCCAAACCTTTGACCTGGGC-3′
TNF-ɑ	F	5′-TTGACCTCAGCGCTGAGTTG-3′
	R	5′-CCTGTAGCCCACGTCGTAGC-3′
IL-6	F	5′-GTACTCCAGAAGACCAGAGG-3′
	R	5′-TGCTGGTGACAACCACGGCC-3′
IL-17	F	5′-TCTCATCCAGCAAGAGATCC-3′
	R	5′-AGTTTGGGACCCCTTTACAC-3′
CCR3	F	5′-CCCGAACTGTGACTTTGCT-3′
	R	5′-CCTCTGGATAGCGAGGACTG-3′
MUC5AC	F	5′-AGAATATCTTTCAGGACCCCTGCT-3′
	R	5′-ACACCAGTGCTGAGCATACTTTT-3′

### Western blot analysis

The detailed method is given in Supplementary information 1.

### Animal experiment and treatment regimen

Male Balb/c mice (6–8 weeks old; 20‒22 g) were obtained from The Jackson Laboratory (Bar Harbor, ME, USA). The mice were housed in a specific-pathogen-free barrier facility at 21 ± 2 °C with a relative humidity of 60 ± 10% under a 12-h light/dark cycle. Feed and water were provided ad libitum. The animal protocol was approved by the committee for animal welfare at Daejeon University (DJUARB2017-024). This study was performed in strict accordance with the Guide for the Care and Use of Laboratory Animals of the National Institute of Health. All animal procedures were conducted in accordance with the guidelines of the Institutional Animal Care and Use Committee of South Korea, Research Institute of Bioscience and Biotechnology (Daejeon, Republic of Korea). Mice were divided into 6 treatment groups (n = 8 for each group): (a) BALB/c normal, (b) CFD-sensitized control mice, (c) positive control- 10 mg/kg montelukast-treated CFD-sensitized mice, (d) 200 mg/kg KG3P-treated CFD-sensitized mice, (e) 100 mg/kg KG3P-treated CFD-sensitized mice, and (f) 20 mg/kg nepetin-treated CFD-sensitized mice. Following the acclimatization period, all groups except group 1 were administered 100 μL of CFD (Coal (5 mg/ml, Fly ash (10 mg/ml), and diesel exhaust particles (DEP; 5 mg/ml) in saline by intratracheal instillation thrice at 3 day intervals for 12 days using bronchial tubes, as previously described^54^. Montelukast, KG3P and nepetin were orally administered daily for 11 days at the above mentioned dosages. On day 12, all mice were euthanized and blood, bronchoalveolar lavage fluid (BALF), and lungs tissues collected for further experiments. The schematic diagram for the experimental protocol is presented in Fig. 3A.

### Collection of bronchoalveolar lavage fluid (BALF) and lung cells

BALF was collected 24 h after the last oral injection of samples. Mice were anesthetized by an i.p. injection of 10% urethane (100 µL; Sigma-Aldrich, Korea). A tracheotomy was then performed, and a cannula inserted into the trachea. Ice-cold DMEM was instilled into the lungs, and BALF collected. Total cell counts were measured with a haemocytometer. For cytological examination, cells were prepared with a Cytospin (Hanil Science, Korea), fixed, and stained with a modified Diff-Quick stain. Differential cell counts were determined using at least 500 cells on each cytospin slide. Blood was collected by cardiac puncture, allowed to clot, then centrifuged; aliquots of serum were stored at − 70 °C for ELISA.

### Flow cytometric analysis

For FACS analysis of lung tissues, enzymatic digestion of the lungs was performed as previously described^55^. Briefly, mice were anesthetized, and the lungs carefully removed. After three washes, the lung tissue was cut into small pieces and transferred to a 15-mL conical tube containing 20 mL of HBSS with 2% FBS (Gibco-BRL, Grand Island, NY) and 1 mM EDTA (Sigma) for 30 min at 20–25 °C. After washing, the lung pieces were incubated with 1 mg/mL collagenase (type IV; Sigma), with shaking. The lung mixture was then filtered through a 70-µm pore size nylon Cell Strainer (BD Falcon, Bedford, MA, USA) and centrifuged for 20 min at 2,000 rpm. The cell pellet was collected, and the cells washed twice. BALF was collected as described in the previous section and thereafter, cells were incubated with monoclonal antibodies (mAbs) against CD3e (145-2C11, hamster IgG), CD4 (RM4-5, rat IgG2a), CD8 (53-6.7, rat IgG2a), CD19 (ID3, rat IgG2a), CD25 (3C7, Rat IgG), and Gr-1 (RB6-8C5, rat IgG2b). All fluorochrome-labelled mAbs and isotype control IgGs were purchased from BD Biosciences (San Diego, CA, USA), and CCR3 (83103, Rat IgG2a) was purchased from R&D system (Minneapolis, MN, USA). Cells from the lungs and BALF were incubated with FITC- and PE-labelled mAbs for 30 min, washed with PBS, fixed with 4% paraformaldehyde (Sigma-Aldrich, Korea) for 20 min, washed with PBS, and then stored at 4 °C until analysis by two-colour flow cytometry on a FACS Caliber (BD Biosciences, Mountain View, CA, USA).

### BALF and cytokine measurements

Mice were anesthetized by an i.p. injection of urethane (100 µL; Sigma-Aldrich, Korea), and their lungs gently lavaged with 1 mL of 0.9% saline via a tracheal cannula. Total and differential BALF cell counts were determined as previously described^56^. Samples were centrifuged at 2,000 rpm for 10 min, and the supernatants stored at − 80 °C. Asymmetric dimethyl-arginine (ADMA), symmetric dimethyl-arginine (SDMA), TNF-α, IL-6, IL-1β, IL-17, CXCL-1, CCR3 and MUC5AC in serum, BALF and lungs tissue were measured by ELISA using a monoclonal antibody-based mouse interleukin ELISA kit (R&D system), according to the manufacturer’s instructions.

### Histological examination

Lungs were infused via the trachea with 1 mL of 10% neutral formalin and immersed in the same fixative for at least 24 h. Tissues were paraffinized, and 6-µm sections were cut and stained with H&E and Mason trichrome staining (both obtained from Sigma-Aldrich) to assess cell infiltration and fibre formation, respectively, under a light microscope. To determine the severity of inflammatory cell infiltration, peribronchial cell counts, extent of mucus production and goblet cell hyperplasia in the airway epithelium were blindly quantified using the 5-point (0–4) grading system described by Tanaka et al*.*^57^ .

### Immunohistofluorescent (IHF) staining

The detailed method is given in Supplementary information 1.

### Statistical analysis

The detailed method is given in Supplementary information 1.

## Supplementary information

Supplementary Information 1.

## Data Availability

The data required for this study is present in this main manuscript file and the Supplementary information 1 respectively.
