# Supplementary material for: Effects of a herbal formulation, KGC3P, and its individual component, nepetin, on coal fly dust-induced airway inflammation
Source: Sci Rep. 2020 Aug 20;10:14036. doi: 10.1038/s41598-020-68965-5 (PMC7441173; doi:10.1038/s41598-020-68965-5)
Supplement: Supplementary file 1 — Supplementary Information 1. [file 41598_2020_68965_MOESM1_ESM.docx]

**Effects of a herbal formulation, KGC3P, and its individual component, nepetin, on coal fly dust-induced airway inflammation**

Evelyn Saba^1^, Young-sil Lee^2^, Won-Kyung Yang^3,6^, Yuan Yee Lee^1^, MinKi Kim^1^,Su-Min Woo^9^, Kil-Soo Kim^1^, Young-Sam Kwon^1^, Tae-Hwan Kim^1^, Dongmi Kwak^1^, Yang-Chun Park^3^, Han Jae Shin^4^, Chang Kyun Han^5^, Jae-Wook Oh^7^, Young Cheol Lee^8^, Hyung-Sik Kang^9^, Man Hee Rhee^1*^, and Seung-Hyung Kim^6*^

^1^ Department of Veterinary Medicine, College of Veterinary Medicine, Kyungpook National University, Daegu 41566, Korea

^2^ Herbal Medicine Research Division, Korea Institute of Oriental Medicine, 1672 Yuseong-daero, Yuseong-gu, Dajeon 34054, Republic of Korea

^3^ Division of Respiratory Systems, Department of Internal Medicine, College of Korean Medicine, Daejeon University, Daejeon, Korea

^4^ KT&G Research Institute, Daejeon 34128, Korea

^5^ KGC Research Institute, Daejeon 34128, Korea

^6^ Institute of Traditional Medicine and Bioscience, Daejeon University, Daejeon 34520, Republic of Korea

^7^ Department of Stem Cell and Regenerative Biotechnology, Konkuk University, Seoul 05029, Republic of Korea

^8^ Department of Herbology, College of Korean Medicine, Sangji University, 83 Sangjidae-gil, Wonju, Gangwon-do 26339, Republic of Korea

^9^ School of Biological Sciences and Technology, Chonnam National University, Gwangju 500-757, Republic of Korea

*Corresponding author at: Laboratory of Physiology and Cell Signalling, College of Veterinary Medicine, Kyungpook National University, Daegu 41566, Republic of Korea (Man Hee Rhee, PhD). Institute of Traditional Medicine and Bioscience, Daejeon University, Daejeon 34520, Republic of Korea (Seung-Hyung Kim, PhD

E-mail: [rheemh@knu.ac.kr](mailto:rheemh@knu.ac.kr) (Man Hee Rhee, PhD), [sksh518@dju.kr](mailto:sksh518@dju.kr) (Seung-Hyung Kim, PhD).

**Supplementary file 1 for materials and methods**

**Chemicals and Reagents.** Dulbecco's modified Eagle's medium (DMEM; Daegu, Korea), Foetal bovine serum (FBS), Dulbecco’s phosphate buffered saline (DPBS; WelGene Co., Korea), streptomycin, and penicillin (Lonza, MD, USA), TRIZOL^®^ reagent (Invitrogen, Carlsbad, CA, USA), Ammonium-Chloride-Potassium (ACK) lysing buffer (Gibco, Life Technologies Cooperation, NY, USA), Bovine serum albumin (BSA) (Thermofisher Scientific, Seoul, Korea) oligo-dT (Bioneer oligo synthesis), iNOS, COX-2, TNF-α, IL-6, IL-1β, IL-17, CCR3, and MUC5AC primers were obtained from Bioneer (Bioneer, Daejeon, Korea). CFA and 3-(4,5-dimethylthiazol-2-yl)-2,5-diphenyltetrazoliumbromide (MTT) were purchased from Sigma-Aldrich (St. Louis, MO, USA). Specific antibodies used against phospho- and/or the total form of ERK, JNK, p38, IKK α/β, IκB, NF-κB p65, iNOS, COX-2, IRAK1, β-actin, and secondary antibody rabbit HRP linked antibody were purchased from Cell Signaling Technology (Danvers, MA, USA). All other reagents and chemicals were obtained from Sigma-Aldrich. CFD used was firstly composed of the phenol compounds (μg/mg): Hydroquinone (0.23), Resorcinol (0.33), Catechol (0.9), Phenol (3.56), m+p Cresol (7.55) o-Cresol (4.42) to obtain the total (16.91). Secondly, it was composed of the aromatic amines (µg/mg): 1-Naphthylamine (1.36), 2-Naphthylamine (1.72), 3-Aminobiphenyl (0.21) and 4-Aminobiphenyl (0.16); third, Benzo alpha pyrene (83.39); and finally, Fly ash (μg/mg), SiO_2_ (4), Fe_2_O_3_ (2.3), AI_2_O_3_ (3.2), CaO (0.3), MgO (0.7), TiO_2_ (0.4), and Ignition loss (0.4).

**Cell culture**. Murine alveolar macrophage cell line MH-S, originating from the American Type culture collection, was cultured in DMEM supplemented with 10% FBS (WelGene Co, Daejeon, Korea) and 100 IU/mL penicillin and 100 µg/mL streptomycin sulphate (Lonza, MD, USA). The incubating conditions were humidified 5% CO_2_ at 37$℃$.

**Nitric oxide (NO) assay**. NO was measured using the method based on the Griess reaction assay. Briefly, MH-S cells were seeded in a 96-well plate and incubated with or without CFA (50 µg/mL) in the absence or presence of KG3P and single compounds at indicated concentrations for 18 h. The cell culture supernatants (100 µL) were mixed with Griess reagent (0.2% naphthylethylenediamine dihydrochloride and 2% sulphanilamide in 5% phosphoric acid) in DDW at equal volumes and incubated for 5 min at 20-25$℃$. The absorbance in each well was then analysed at 540 nm in microplate reader (Versamax, Microplate Reader, Molecular devices, CA, USA).

**Cell Viability (MTT) assay.** To determine the cytotoxic effects of samples, a cell viability assay was performed using 3-(4, 5-dimethylthiazol-2-yl)-2, 5-diphenyltetrazolium bromide reagent which was added to the culture medium at a final concentration of 0.1 mg/mL. After 4 h of incubation at 37$℃$ in 5% CO_2_, the resulting violet-coloured crystals were dissolved in 100 µL/well dimethyl sulfoxide (DMSO) and the absorbance measured at 560 nm.

**RNA extraction and Polymerase chain reaction (PCR)**. MH-S cells were pre-treated with or without KG3P and single compounds at indicated concentrations for 30 min, followed by stimulation with CFA (50 µg/mL) for 18 h. RNA was collected from cells and lung tissue using TRIZOL^®^ reagent (Invitrogen, Carlsbad, CA, USA) following the manufacturer’s instructions. Total RNA (2 µg) was annealed with Oligo-dT (Bioneer Co, Daejeon) for 10 min at 70$℃$, cooled for 5 min on ice, reverse transcribed using reverse transcriptase pre-mix (Bioneer Co., Daejeon) in 20 µL of reaction mixture and ran for 90 min at 42.5$℃$ on a thermocycler. To inactivate the reverse transcriptase, the reaction was terminated at 95$℃$ for 5 min. The reverse transcription polymerase chain reaction (RT-PCR) was performed using aliquots of cDNA obtained from RT reaction in a PCR premix (Bioneer Co., Daejeon). The PCR products were then electrophoresed on 1% agarose gel stained with ethidium bromide and visualized using Image quant LAS 500 (GE Health Care Life Sciences, Seoul, Korea). The intensity of band densities was normalized to GAPDH, a housekeeping gene used as the RNA internal standard, and the ratios compared. Real-Time PCR was performed using SYBR green fluorescence. PCR primer sequences are listed in Table 1.

**Western blot analysis**. MH-S cells were either not treated or treated with KG3P and single compounds in the presence or absence of CFA (50 µg/mL). Proteins were collected from cells and from the lung tissue of mice with CFA-induced airway inflammation using cytosolic and nuclear proteins commercially available kit (NE-PER^®^ Nuclear and cytosolic extraction reagents; Thermo scientific, Seoul, Korea). Proteins were then measured using the PROMEASURE assay kit (PRO-PREP, iNtRON Biotechonology), separated by 10% SDS-PAGE and transferred onto PVDF membrane (Millipore, Immobilion ^®^-P, Billerica MA, USA). Nonspecific binding on the nitrocellulose filter paper was minimized with a blocking buffer containing 5% non-fat dry milk and 0.1% Tween-20 in TBS. The membranes were then incubated with specific primary antibodies overnight at 4$℃$ followed by a 1 h incu­bation with horseradish peroxidase-conjugated anti-rabbit antibody (1:3000 dilution, Cell Signaling) at 20-25$℃$. Bound antibodies were visualized using enhanced chemiluminescence (Supex, Daegu, Korea) and images analysed using ImageJ software. β-actin was used as the internal control.

**Immunohistofluorescent (IHF) staining.** The lung tissues were frozen at –20$℃$, and sections cut to a thickness of 20 µm using a Cryostat Microtome (CM 3050S, Leica Microsystems, Wetzlar, Germany). Lung sections (20 µm) were fixed with 4% paraformaldehyde and 4% sucrose in phosphate buffered saline (PBS) at 20-25$℃$ for 40 min, permeabilized with 0.5% nonidet P-40 in PBS, and blocked with 2.5% horse serum and 2.5% bovine serum albumin for 16 h at 20-25$℃$. Double immunofluorescence staining was performed by incubating tissue sections with antibodies for IRAK-1 (Cell Signalling Technology, Inc. USA) overnight at 4$℃$. Subsequently, fluorescein-conjugated secondary antibody was added for 2 h, and nuclear staining performed using DAPI. Sections were observed using an Eclipse T*i*-E inverted fluorescent microscope (Nikon Instruments Inc., Mississauga, Canada).

**Statistical analysis.** Data were analysed by one-way analysis of variance (ANOVA) or unpaired Student’s *t*-test followed by Dunnett’s multiple comparison test using SPSS version 14.0. The results are presented as mean *±* SEM, and significant differences denoted as: *#p* <0.05, *##p* <0.01, and *###p* <0.001 (compared to WT), and **p* <0.05, ***p* <0.01, and ****p* <0.001 (compared to CFD (and CFA for *in vitro* studies)).

**Supplementary file 1 for figure legends**

The full length blots for the gel images are given below in the pictures.

Supplementary figure S2a= MAPK pathway in MH-S cells

Supplementary figure S2b= NF-κB pathway in MH-S cells

Supplementary figure S2c= NF-κB pathway in lungs tissue

Supplementary figure S2d= MAPK pathway in lungs tissue

Supplementary figure S3= Cell population for FACS in Lung and BALF tissue.

**
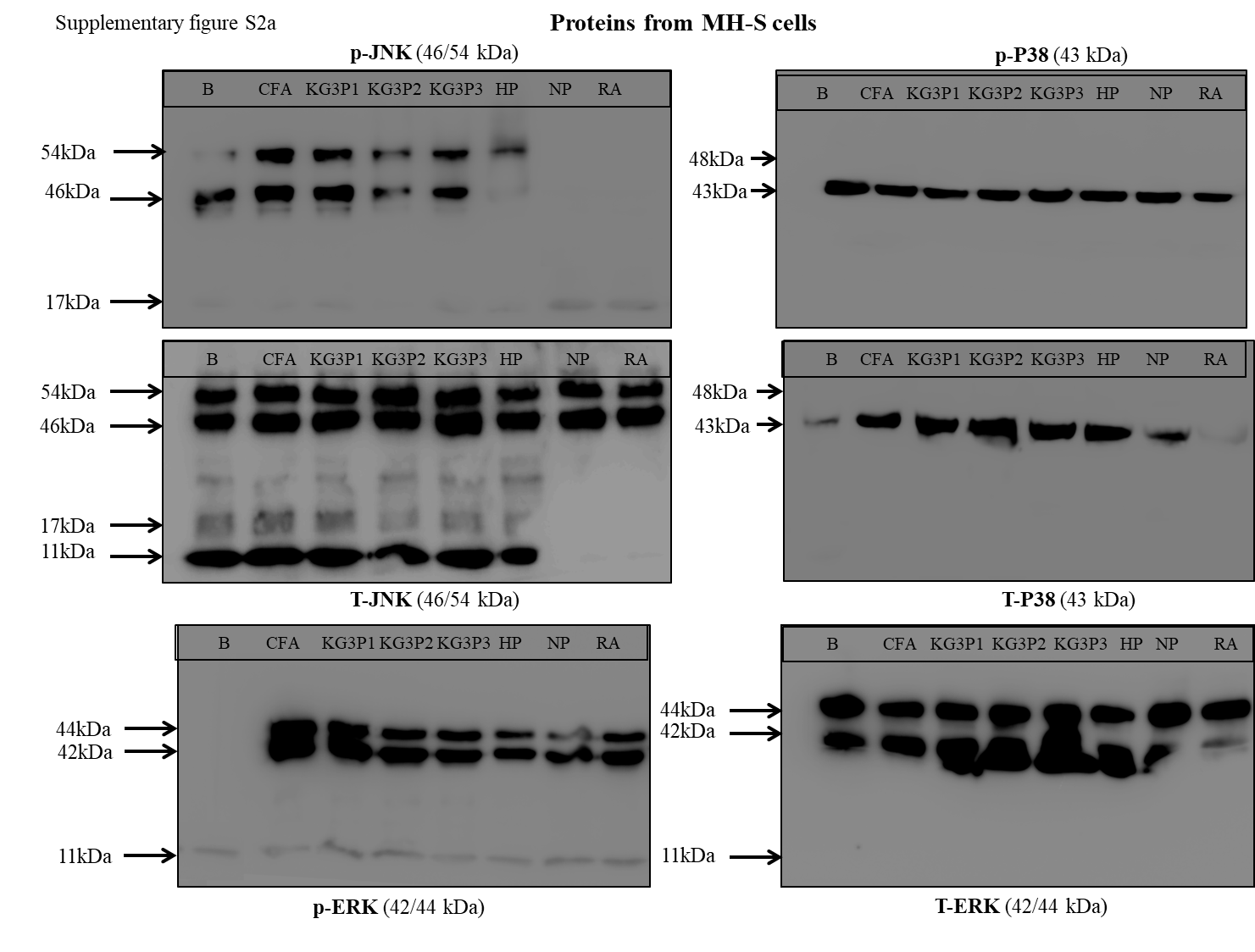
**

**
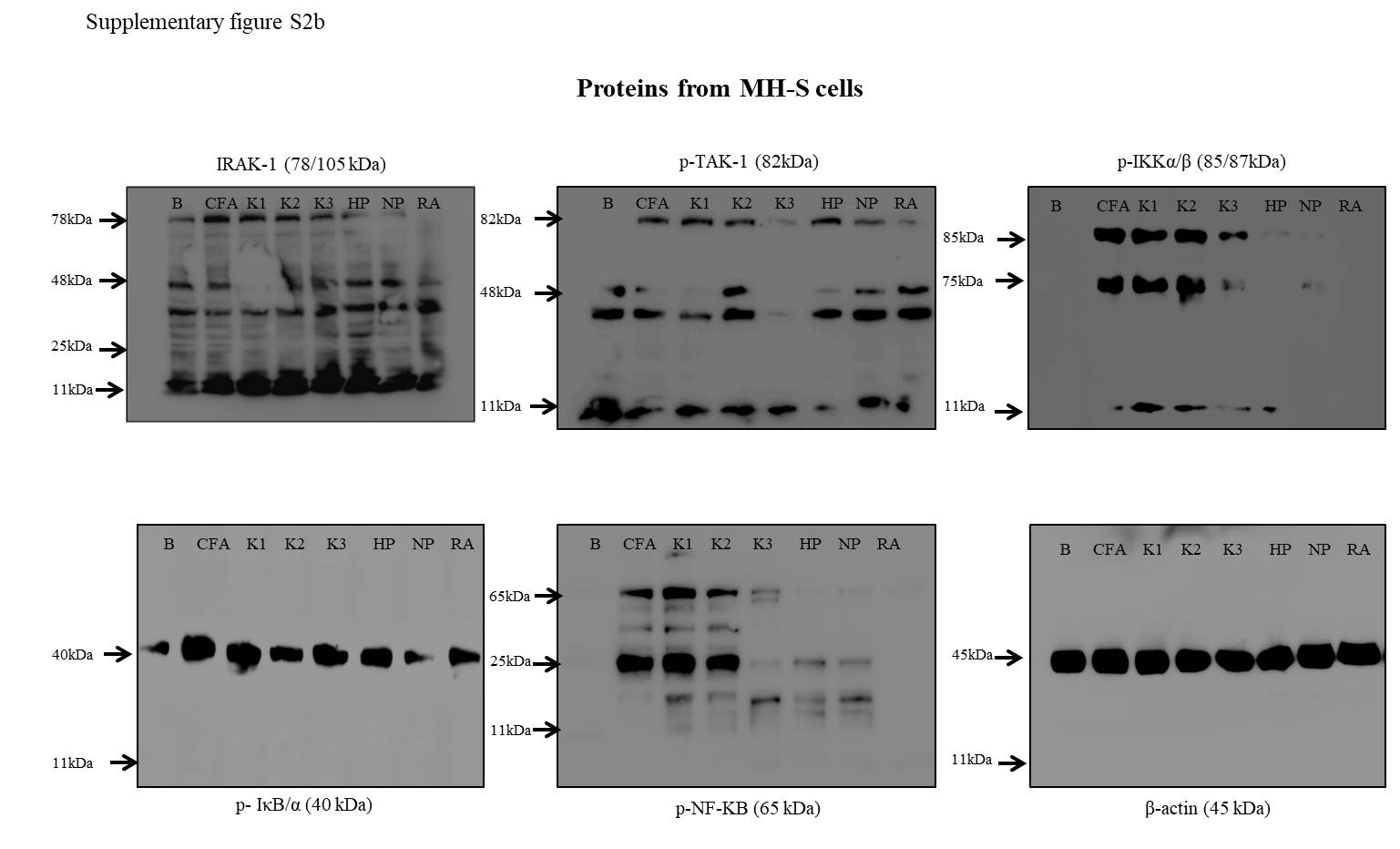
**

**
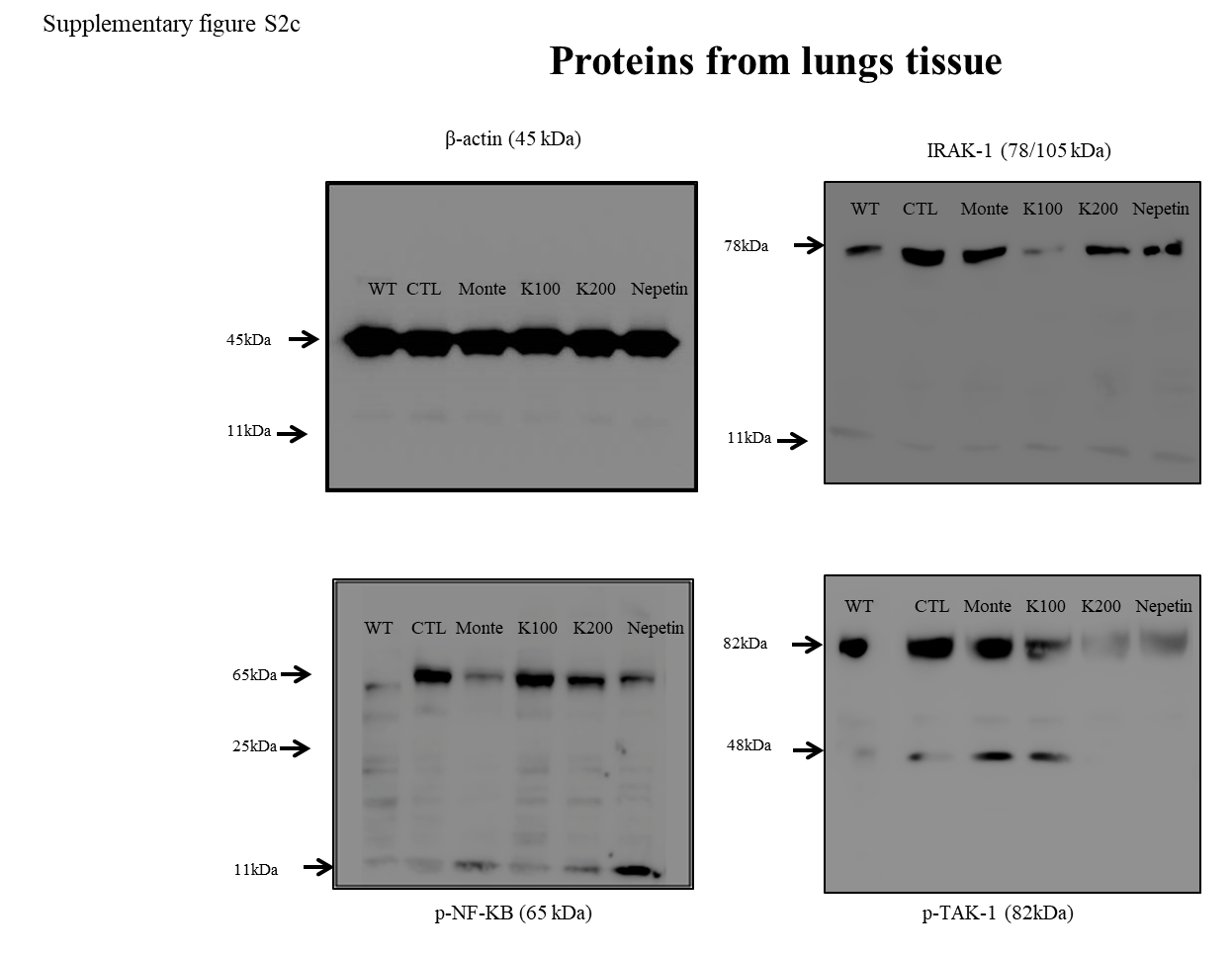
**

**
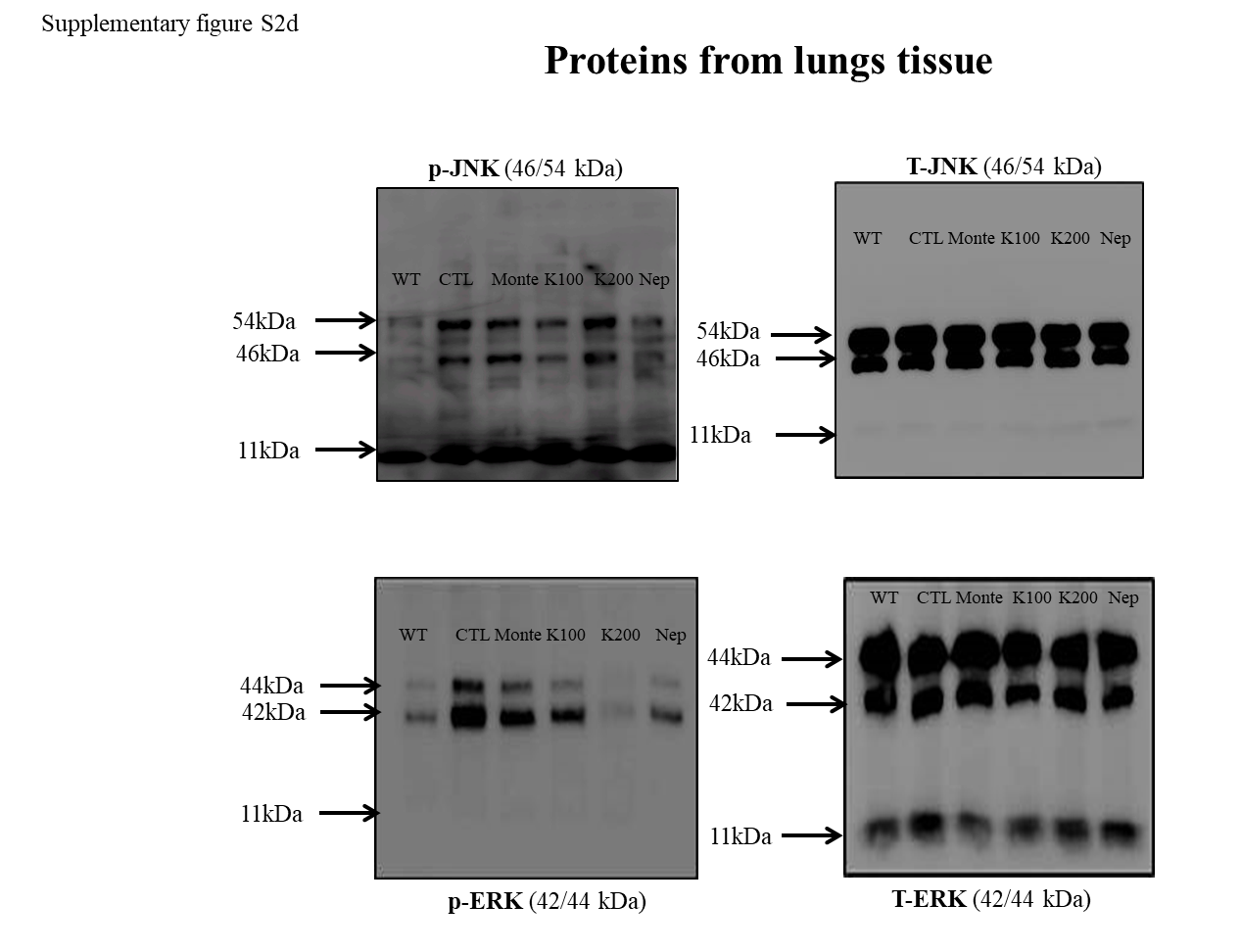
**

**
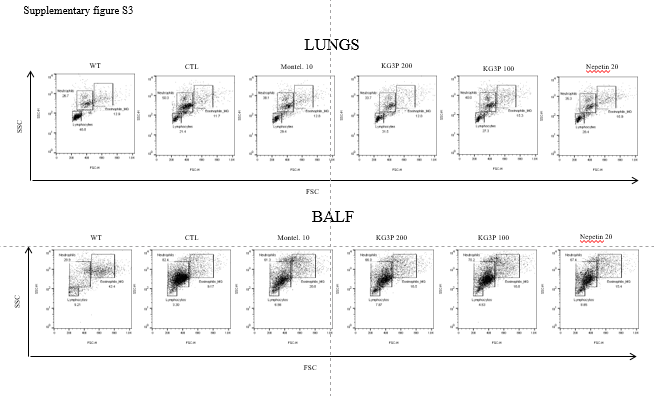
**
